# Visual Loop-Mediated Isothermal Amplification (LAMP) Assay for Rapid On-Site Detection of *Escherichia coli O157: H7* in Milk Products

**DOI:** 10.3390/foods13132143

**Published:** 2024-07-05

**Authors:** Shuangshuang Cui, Yong Wei, Can Li, Jian Zhang, Yunfeng Zhao, Xiayu Peng, Fengxia Sun

**Affiliations:** 1 School of Food Science and Technology, Shihezi University, Shihezi 832000, China; alexandra9982022@163.com (S.C.); li1821632098@163.com (C.L.); zhangjian0411@163.com (J.Z.); yunfeng_zhao@shzu.edu.cn (Y.Z.); 2 Key Laboratory for Food Nutrition and Safety Control of Xinjiang Production and Construction Corps, School of Food Science and Technology, Shihezi University, Shihezi 832000, China; 3 Xinjiang Tianrun Dairy Co., Ltd., Wuchang Road No. 2702, Urumqi 830000, China; 17302227693@163.com; 4 Key Laboratory of Agricultural Product Processing and Quality Control of Specialty (Co-Construction by Ministry and Province), School of Food Science and Technology, Shihezi University, Shihezi 832000, China; 5 College of Animal Science and Technology, Shihezi University, Shihezi 832000, China

**Keywords:** loop-mediated isothermal amplification, *Escherichia coli O157:H7*, visualization detection, immunochromatographic test strips, colorimetric method

## Abstract

(1) Background: Rapid on-site testing is an effective method for the detection of *Escherichia coli O157: H7*(*E. coli O157: H7*) in food ingredients and the environment. (2) Methods: In this study, we developed colorimetric loop-mediated isothermal amplification (LAMP) and immunochromatographic test strips (ICTs) for the rapid and visual detection of *E. coli O157: H7*. This study designed new specific LAMP primers for *E. coli O157: H7* virulence island genes. After the LAMP amplification, the double-stranded DNA target sequence labeled with digoxin and fluorescein isothiocyanate (FITC) at both ends was bound to the anti-digoxin antibody on the gold nanoparticles. Subsequently, it was further bound to the anti-FITC antibody at the T line of the ICTs, forming a positive test result. Hydroxynaphthyl blue dye was directly added to the LAMP amplification product. A blue color indicated positive results, while a purple color indicated negative results. (3) Results: Two visualization methods showed high specificity for the target strains. The visualization tests had sensitivities of 5.7 CFU mL^−1^, and the detection limit of the *Escherichia coli O157: H7* in artificially contaminated milk samples was 5.7 × 10^2^ CFU mL^−1^, which was consistent with the results of the standard method (LAMP-electrophoresis method) used in commercial inspection. (4) Conclusions: Both methods could be useful in remote and under-resourced areas.

## 1. Introduction

*Escherichia coli* is a notorious foodborne pathogen that frequently contaminates meat and vegetables [1,2]. *Escherichia coli O157: H7* (*E. coli O157: H7*) is the most common serotype and can cause acute gastroenteritis, hemorrhagic colitis (HC), and hemolytic uremic syndrome (HUS) in humans worldwide [3,4]. *E. coli O157:H7* can be transmitted in a variety of ways during food processing. It can parasitize domestic animals, such as cows, pigs, chickens, sheep, and dogs, and it can also contaminate meat, dairy, and egg products, vegetables, and fruits [5,6,7]. Dairy cattle are considered to be the most important animal hosts of *E. coli O157: H7*, thus contaminating ground beef, milk, and other foods [8]. The bacteria can be detected in contaminated ground beef that has been frozen at −20 °C for 9 months [9,10]. Therefore, a rapid and easy-to-perform method for the detection of *E. coli O157: H7* is needed to effectively minimize the incidence of contaminated food reaching consumers and to control related foodborne outbreaks.

Microbial culture methods, polymerase chain reaction (PCR) methods [11], electrochemical sensors [12], immunological detection techniques [13], etc., can detect *E. coli O157: H7*, but these methods are limited by the constraints of laboratory instrumentation and operators, and they are unable to detect *E. coli O157: H7* in a timely and effective manner in remote areas where resources are limited [14]. With the rapid development of molecular diagnostic technology, some isothermal amplification methods have been developed and applied to the detection of pathogenic bacteria. The loop-mediated amplification (LAMP) technology [15] is representative of isothermal techniques and has been developed into a mature system. It uses four to six specially designed primers to ensure highly specific results, and can produce about 10^3^ times more amplification products than the conventional PCR in a short period of time after heating in a simple water bath, which has the advantages of saving time and having a low cost. Due to its superior performance, the LAMP method has been demonstrated to be a reliable and robust method for the detection and identification of viral and microbial pathogens [16]. The performance of the LAMP, VIDAS UP, and ISO 16654 (International Organization for Standardization 16654) [17] methods for the detection of *E. coli O157: H7* in different food matrices was evaluated, and LAMP showed a higher specificity and sensitivity than the ISO 16654 and VIDAS UP methods, and it took less time to perform [18].

LAMP-amplified DNA products can be detected by a variety of methods, such as visual inspection, agarose gel electrophoresis (AGE), colorimetry, electrochemical detection, and immunochromatographic test strip (ICT) methods [19]. Colorimetric agents and ICTs are commonly used visualization methods for rapid on-site detection and are simple to operate. Metal ion indicators such as calcein and hydroxynaphthol blue (HNB) do not affect the process of the LAMP reaction, but the amount of metal ions is strictly required, and unclear positives will occur if there are too many of them. Immunochromatographic test strips are an efficient and precise method applicable to on-site detection [20]. They have the advantages of a simple operation and good detection performance, and do not require specific instruments. In recent years, there has been a gradual increase in the number of methods for the detection of microorganisms in foodstuffs using immunochromatographic test strips because of their low cost, simple manufacturing methods, and portability benefits [19,20,21,22].

The objective of this study was to create a quick and straightforward technique for distinguishing *E. coli O157: H7*. This study designed and screened LAMP primers against the *E. coli O157: H7* virulence island gene through comparative genomic analysis. Colorimetric LAMP and immunochromatographic methods were established to distinguish *E. coli O157: H7* from other pathogenic bacteria. These assays were then combined with direct DNA extraction methods for the on-site detection of pathogenic bacterial species.

## 2. Materials and Methods

### 2.1. Reagents and Materials

*Listeria monocytogenes* (ATCC19115), *Escherichia coli O157: H7* (CICC21530), *Salmonella typhimurium* (ATCC14028), *Staphylococcus aureus* (ATCC25923), and *Campylobacter jejuni* (BW180151), used in the present experiments, were obtained from the Xinjiang Academy of Reclamation Sciences (Shihezi, China).

Bst-DNA polymerase large fragment enzyme (8 U/μL), MgSO_4_ (100 mM), and ThermoPol buffer (10×) were obtained from Nanjing Vazyme Biotech Co., Ltd. (Jiangshu, China). Sample pads (SB08), binding pads (RB45), NC membrane (CN140), absorbent paper (CH27), and base plates (SM31-25) were obtained from Shanghai Kinbio Tech Co., Ltd. (Shanghai, China). The dNTPs (no. B500056), ddH2O (no. B541017), and agarose (no. A620014) were obtained from Sangon Biotech Co., Ltd. (Shanghai, China). Hydroxynaphthol Blue (HNB, H103107) and Bovine serum albumin (BSA, B265993) was provided by Shanghai Aladdin Biochemical Technology Co., Ltd. (Shanghai, China). Goat anti-mouse IgG (BF03001) was purchased from Suzhou Biodragon Technology Co., Ltd. (Suzhou, China). Anti-digoxin antibody was obtained from Wuxi Determine Biotechnology Co., Ltd. (Wuxi, China), and anti-FITC antibody (901035) was obtained from Guangzhou Greilin Biotechnology Co., Ltd. (Guangzhou, China). All of the solutions were prepared with ultrapure water (resistivity: 18.2 MΩ-cm).

### 2.2. Strain Culture and DNA Extraction

One inoculation loop of the bacterial solution (about 5 µL) was added to 4 mL of Brain–Heart Infusion (BHI) liquid medium and mixed. The mixture was then incubated on a shaker at 37 °C for 12 h. After incubation, the bacterial solution was streaked on Plate Count Agar (PCA) medium and incubated for 12-18 h. Single colonies were selected from the plates and transferred to the BHI liquid medium. The mixture was thoroughly mixed and incubated at 37 °C for 12 h. Then, 100 μL of the incubated bacterial solution was obtained and diluted tenfold with saline. Four gradients from 10^−3^ to 10^−6^ were selected for smear counting. The remaining bacterial solution was then boiled to extract the DNA.

The DNA was extracted by the boiling method based on TE buffer (Tris-EDTA buffer), with minor modifications. An amount of 1 mL of the bacterial culture was centrifuged at 9800× *g* and boiled in 100 μL of the TE buffer (pH of 8.0) for 10 min, and then centrifuged at 8000× *g* for 5 min. The template DNA was obtained by collecting the supernatant, and then stored at −20 °C for spare use. The concentration of DNA was measured by the Nanodrop 2000 (Shanghai Zaitou Biotechnology Co. Ltd., Shanghai, China), and the purity of the DNA was measured by 2% agarose gel electrophoresis at 120 V and 90 mA for 30 min.

### 2.3. LAMP Primer Design

The conserved sequence of *rfbE* (GenBank ID: AF163332.1) and the virulence *stx2* gene (GenBank ID: MF039302.1) of *E. coli O157: H7* was used as the target gene, and highly conserved sequences of the target gene were identified using the BLAST program. The LAMP primers were designed using primer design software Primer Explorer V5 (http://primerexplorer.jp/e/index.html (accessed on 20 April 2022)), and the BLAST program was used to validate the specificity of the primers. Primer screening was conducted using the same amplification conditions and analyzed by agarose gel electrophoresis (AGE). The sequences of the functional primers used to visualize the LAMP are shown in Table 1. The primers were synthesized and modified by General Bio Co., Ltd. (Chuzhou, China).

### 2.4. LAMP System Optimization

We then optimized the LAMP amplification system. The reaction system was optimized for Mg^2+^ (concentrations of 2 μM, 4 μM, 6 μM, 8 μM, and 10 μM, respectively), dNTP concentrations (concentrations of 0.8 μM, 1.2 μM, 1.6 μM, 2.0 μM, and 2.4 μM, respectively), the ratio of internal and external primer concentrations (ratios of internal and external primer concentrations of 1:1, 2:1, 3:1, 4:1, 6:1, and 8:1, where the concentration of the external primer was 0.2 μM), the Bst-DNA polymerase enzyme concentrations (0.08 U/μL, 0.16 U/μL, 0.24 U/μL, 0.32 U/μL, 0.40 U/μL, and 0.48 U/μL, respectively), the reaction temperature (61 °C, 63 °C, 65 °C, 67 °C, and 69 °C), and the reaction time (35 min, 40 min, 45 min, 50 min, and 55 min). The optimization was carried out to select the best reaction system and perform the specificity and sensitivity tests.

### 2.5. Feasibility Detection

Feasibility is a requirement for establishing and using experimental methods. Both the colorimetric LAMP and LAMP-ICTs were used to perform the visualized LAMP reactions. A colorimetric LAMP analysis was conducted by adding an appropriate concentration of HNB dye to 10 μL of the LAMP product to observe the results. The LAMP-ICT method utilizes the specific binding of the T line coating and modified primers to visualize the results. A sample of the LAMP product was diluted and then assayed using the LAMP-ICTs.

### 2.6. Optimization of Dye Concentration

To determine the dye concentration with the most pronounced color contrast, we tested a range of HNB dye concentrations, including 0.4 mM, 0.6 mM, 0.8 mM, 1.0 mM, and 1.2 mM. Based on visual observation, we selected the concentration that produced the most significant color difference between the negative and positive controls.

### 2.7. Selection of Gold Nanoparticle Size

Gold nanoparticles (AuNPs) of three sizes were synthesized using the trisodium citrate reduction method. Briefly, the conical flasks were soaked in aqua regia overnight and vaporized three times in ultrapure water. An amount of 0.5 mL of 1% HAuCl_4_ was added to a conical flask containing 49.5 mL of sterile distilled water. After boiling and stirring, we added different volumes of trisodium citrate and continued to heat it for 15 min. After cooling to room temperature, we added ultrapure water to bring the volume up to 50 μL, poured it into a brown bottle, and stored it at 4 °C.

### 2.8. Optimization of LAMP-ICTs Assay

In addition to the AuNPs, the preparation conditions of the ICTs have an important effect on the sensitivity. First, gold-labeled antibody conjugates were prepared by electrostatic adsorption. Prior to the conjugation, volumes (0, 2, 4, 6, 8, and 10 μL) of 0.1 M K_2_CO_3_ were optimized to adjust the optimal pH of the AuNPs. Digoxin antibody additions (2.5, 3.5, 4.5, 5.5, and 6.5 μg), T line encapsulant concentrations (1.2, 1.5, 2.0, 2.2, 2.5, and 3.0 mg mL^−1^), and LAMP product upsampling volumes (1, 2, 3, 4, 5, and 6 μL) were optimized. The stability of the gold-labeled antibody was verified by 10% Nacl. The optimal results of the T line encapsulant concentration and the LAMP product upsampling volume were verified by the test strip T line color development.

### 2.9. Specificity and Sensitivity Detection in the Bacterial Solution

Five bacteria with different properties (*Listeria monocytogenes* (ATCC19115), *E. coli O157: H7* (CICC21530), *Salmonella typhimurium* (ATCC14028), *Staphylococcus aureus* (ATCC25923), and *Campylobacter jejuni* (BW180151)) were used to perform the method-selective assay. Sterile saline was used to make 10-fold multiplicative dilutions of the bacterial fluids, and DNA from different concentrations of bacterial fluids was extracted separately as templates to assess the detection limits. The LAMP-AGE, colorimetric LAMP, and LAMP-ICT methods were used to evaluate the specificity and the limit of detection (LOD).

### 2.10. Detection of E. coli O157: H7 in Milk Samples

During the production process, milk can be contaminated by various pathogenic bacteria, which can make the testing more complex. Before the testing, the samples were analyzed using the standard culture method (GB 4789.6–2016, China) [25] to confirm the absence of *E. coli O157: H7*. Next, 100 μL of a pathogenic bacteria solution was added to 900 μL sterile milk samples for artificial contamination experiments. To test the specificity of the two visualization methods in the milk samples, a mixed contamination of aseptic milk samples with *E. coli O157: H7* and other strains was simulated to mimic actual contamination. Tenfold dilutions of artificially contaminated samples were prepared by adding different concentrations of *E. coli O157: H7* bacterial fluids to aseptic milk samples for the sensitivity detection of two visualization methods in the milk samples. Additionally, the milk samples were added and recovered at various concentrations to confirm the quantification ability and stability of the LAMP-ICTs. All of the experiments were repeated three times to ensure the reproducibility.

## 3. Results and Discussion

### 3.1. Primer Screening

For the two primers designed for the *E. coli O157: H7 stx2* gene, the 21 primer could not recognize the DNA chain, and the DNA band can be seen in the gel electrophoresis band at the same position as the DNA band in the NC. Primer 22 was found to be suitable for the LAMP detection of *E. coli O157: H7*. However, under the same conditions, the two primers (E1 and E2) designed for the rfbE gene were unable to amplify the bands (Figure S1). Therefore, we selected 22 primers for the LAMP amplification experiments.

### 3.2. Optimization of LAMP Reaction

The LAMP reaction was analyzed by agarose gel electrophoresis in order to obtain the optimal LAMP amplification system (Figure S2). Betaine has the effect of reducing the Tm value, and during the optimization process, it was found that the amplification efficiency of the *E. coli O157: H7* system with the addition of betaine was lower, or even unable to amplify. This may be due to the low Tm value of the designed primers. The best amplification results were obtained with the addition of 6 μM of MgSO_4_ and 1.2 μM of the dNTPs. Bright and stable bands were observed when the ratio of the internal to external primers was 4:1 (0.8 μM:0.2 μM). The LAMP system was unable to amplify the bands when the concentration of Bst-DNA polymerase enzyme was lower than 0.16 U/μL. The optimal concentration of the Bst-DNA polymerase was found to be 0.32 U/μL. Clear bands were observed after 40 min of reaction time. The optimal temperature for the LAMP was found to be 63 °C. At 69 °C, the Bst-DNA polymerase was inactivated, leading to reduced amplification efficiency.

### 3.3. Visual Detection Principle

**Figure 1:**
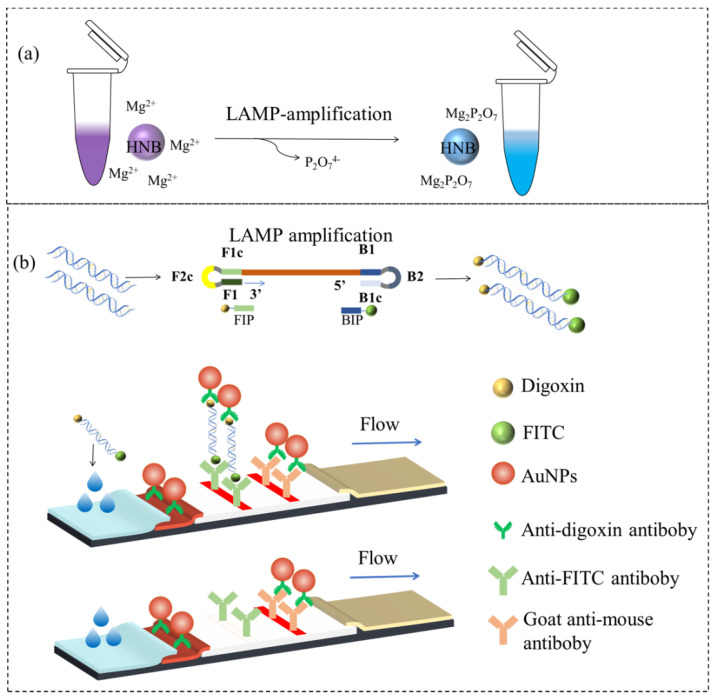
Colorimetric LAMP (**a**) and LAMP-ICT (**b**) detection principle.

The colorimetric LAMP assay principle is shown in Figure 1a. The combination of HNB and Mg^2+^ made the initial color of the reaction system purple; as the LAMP reaction progressed, the Mg^2+^ reacted with the precipitated pyrophosphate ions to form magnesium pyrophosphate precipitate, and the HNB lost the magnesium ions to make the color of the system change to azure blue, while the unreacted system still retained the purple color.

The LAMP-ICT assay principle is shown in Figure 1b. At the end of the LAMP amplification reaction, a double-stranded DNA product was produced that was labeled with FITC on one end and with digoxin on the other. AuNPs were used as markers to label the anti-digoxin antibodies for the preparation of the gold-labeled antibody probes. The ICT test line was coated with the anti-FITC antibody, while the quality control line was coated with the goat anti-mouse IgG. The T and C lines indicate the test and control lines, respectively. When *E. coli O157: H7* was present in the sample, digoxin at one end of the LAMP amplification product bound to the gold-labeled probes on the binding pad, forming a gold-labeled antibody-LAMP product complex. When the complex was chromatographed to the T line by capillary action, it became immobilized to the T line by binding the FITC on it to the FITC antibody on the T line. This resulted in the formation of a red band on the T line. The excess gold-labeled antibody was chromatographed until it reached the C line. It was then bound by the goat anti-mouse IgG. Subsequently, both the T and C lines showed a red band, indicating a positive result. If the sample does not contain *E. coli O157: H7*, the LAMP product will not exist. As a result, the T line will not show any color because it cannot capture colloidal gold. If C line is not colored, the ICTs test result is invalid and the test must be repeated.

### 3.4. Feasibility Detection

The three methods verify each other’s feasibility, and the test results are shown in Figure S3. Figure S3a shows the LAMP electrophoresis; it can be seen that the contrast between the negative and positive results is obvious, and a waterfall band was formed in the positive results. Figure S3b shows the visualization of the detection of the addition of 0.8 mM of the HNB dye, with blue in the positive amplification products and violet in the negative product tubes; Figure S3c shows the schematic diagram of the results of the immunochromatographic test strip detection. The negative result is indicated by color only in the C line, while the T line remains colorless. In contrast, the positive result is indicated by color in both the C and T lines of the test strip. The color difference was most noticeable when viewed with the naked eye at a dye concentration of 1.0 mM after optimizing the dye concentration, as shown in Figure S4.

### 3.5. Selection of AuNPs with Different Particle Sizes

**Figure 2:**
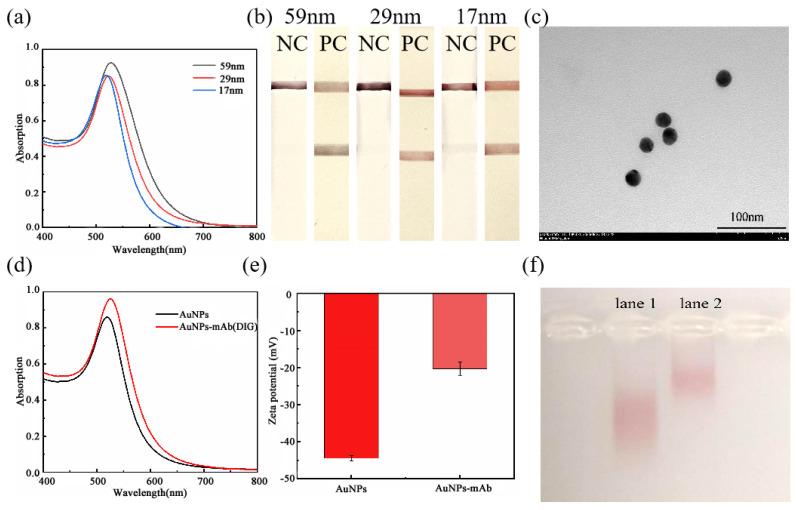
Selection of the AuNP particle sizes. (**a**) UV–Vis spectra of three particle sizes of the AuNPs. (**b**) ICT results for three particle sizes of the AuNPs. (**c**) Transmission electron micrograph of the AuNPs (×100 K). (**d**) UV–Vis spectra of the AuNPs and gold-labeled antibody conjugates. (**e**) Zeta potential of the AuNPs and gold-labeled antibodies. (**f**) AGE of the AuNPs and gold-labeled antibody conjugates (lane 1: AuNPs; lane 2: gold-labeled probes).

Three different particle sizes of AuNPs were prepared by adding 0.7 mL, 0.9 mL, and 1.1 mL of trisodium citrate solution to 50 mL of chloroauric acid solution. The maximum absorption peak wavelengths of the AuNPs with the three different particle sizes were 525 nm, 522 nm, and 520 nm, respectively (Figure 2a). The particle sizes were determined to be 59 nm, 29 nm, and 17 nm using the linear regression equation Y_particle size_ = 6.0308 × λ_max_ − 3118.6. The maximum absorption wavelength decreases and the color of the AuNPs becomes redder as the amount of trisodium citrate increases, while the particle size of the AuNPs decreases. When the AuNPs were applied to the ICTs (as shown in Figure 2b), the 59 nm AuNPs resulted in a purple color, while the negative control of 17 nm of AuNPs showed a false-positive phenomenon, which indicates that non-specific recognition may have occurred. Transmission electron microscopy scans of 29 nm of AuNPs showed that the particle size of the AuNPs was uniform, with a size of approximately 23 nm (Figure 2c).

Both the AuNPs and the digoxin gold-labeled antibody conjugates were scanned at a UV wavelength (400–800 nm), and the results are presented in Figure 2d. Following the coupling of the AuNPs with the digoxin antibody, the maximum absorption peak wavelength shifted from 522 nm to 525 nm, with the redshift phenomenon indicating a successful coupling between the AuNPs and the antibody. The successful coupling of the AuNPs with digoxin antibody is also illustrated by the changes in the zeta potential and migration rate observed in the AGE.

AuNPs carry a negative charge. The antibodies were coupled by electrostatic adsorption, which increased the potential of the coupler due to the positive charge carried by the digoxin antibody (Figure 2e). Figure 2f shows the characterization map of the AGE for both the AuNPs and the digoxin gold-labeled antibody conjugate. Lane 1 represents the AuNPs, while lane 2 represents the digoxin gold-labeled antibody conjugate. Molecules of different sizes have different resistances to migration in the AGE. Therefore, by having different migration speeds during the electrophoresis, it is possible to distinguish between substances of different molecular sizes. As shown in Figure 2f, the migration rate after the conjugated antibody is significantly slower than that of the AuNPs. This indicates that the AuNPs were successfully coupled to the digoxin antibody.

### 3.6. Optimization of ICTs Conditions

In addition to the AuNPs, different conditions of the gold-labeled antibody and test paper also affect the sensitivity of the assay results. First, the optimal pH of the AuNPs was selected by adjusting the volume of the K_2_CO_3_ added. As shown in Figure S5, the color of the liquid darkened after the addition of 10% NaCl and after the addition of 0, 2, and 4 μL K_2_CO_3_. This may be due to the change in the pH of the solution, which caused the AuNPs to self-assemble and become deposited. It should be noted that the pH of all reagents during the gold-labeled probe preparation will affect the color development. This difference can be observed more clearly on the ICTs (Figure S6a), where the signal peak appeared in the T line of the test strip after the addition of 6 µL K_2_CO_3_.

Different amounts of digoxin antibodies were added to the solution of the AuNPs for the incubation of the probe. The brightness of the solution color increased with the increasing antibody concentration (Figure S5). The increasing number of gold-labeled probes in the solution thus improved the stability of the solution. As the number of probes recognized by the T line coating increased, the T line color gradually deepened (Figure S6b), with the best effect being achieved at 5.5 µg. As the antibody addition continued to 6.5 µg, the stability decreased and the T line color decreased (Figure S6b). An excessive addition of the antibody will result in the waste of raw materials and a higher cost. Therefore, we chose to add 5.5 µg of the antibody for incubation.

The concentration of the T line coating and the amount of product added influence the binding of the amplification product to the T line coating. In one-way experiments, the T line did not show any color when the concentration of the FITC antibody coated on the T line was less than 1.2 mg mL^−1^. A low concentration of the encapsulated antibody can lead to the inability to recognize and capture the LAMP product during chromatography. This can cause the T line to appear colorless or light-colored, which reduces the sensitivity. The T line exhibited the highest brightness at a concentration of 2.2 mg mL^−1^ (Figure S6c). Therefore, this concentration was chosen for the T line coating. The experiment involved mixing different volumes of amplification products with the sampling buffer. According to the study results, the T line was colored in 1 µL of the amplification product, but the color was lighter than the other volumes of the amplification product (Figure S6d). It was observed that the gold-labeled probe was not completely consumed by the amplification product at this point. As a result, 2 µL was used as the addition volume in subsequent experiments.

### 3.7. Analysis Performance for E. coli O157:H7

The optimized conditions were used to evaluate the performance of the two visual detection methods. Specificity is a key factor in the practical application of the assay. After completing the LAMP amplification, the color of the HNB dye induced by the product changed to azure blue in the *E. coli O157: H7*, while other tubes showed a blue–violet color (Figure S7b). In the tubes of the other strains, the production of the primer dimer depleted the Mg^2+^, resulting in varying degrees of the purple coloration of the HNB dye. Waterfall bands were observed only in the DNA extracted from the *E. coli O157: H7* by the AGE analysis (Figure S7a). The amplification products were analyzed using the LAMP-ICTs. The T line of the ICTs corresponding to the target strain exhibited color, while the T line of the other strips did not (Figure S7b).

To determine the detection limit of the method, DNA was extracted using a 10-fold dilution of the *E. coli O157: H7* bacterial solution using sterilized saline. This was performed to ensure that the results of the method matched the concentration of the bacterial solution.

**Figure 3:**
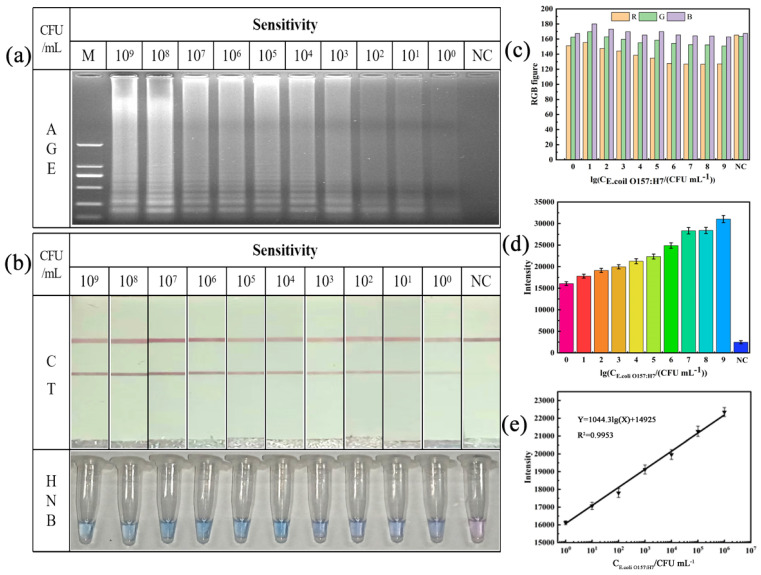
Sensitivity detection of methods. (**a**) AGE detection results. (**b**) LAMP-ICT and colorimetric LAMP detection results. (**c**) RGB data analysis for colorimetric LAMP. (**d**) T line optical intensity analysis of LAMP-ICTs. (**e**) Calibration curve for LAMP-ICT results. M: DNA Marker 2000; NC: negative control.

Three methods, the AGE imaging condition (Figure 3a), colorimetric LAMP, and LAMP-ICTs (Figure 3b), were used to observe the detection limit of the amplification product. Figure 3a shows that, as the concentration of the DNA template decreased due to dilution of the bacterial solution, the electrophoretic brightness decreased in a hierarchical manner. The addition of an appropriate concentration of the HNB dye to the amplification product resulted in a sky-blue color for the amplification result of 10^9^ CFU mL^−1^. As the concentration of the bacterial solution decreased, the color in the HNB tube changed from sky blue to dark blue, and eventually changed to blue–violet when the concentration of the bacterial solution was less than 10^3^ CFU mL^−1^ (Figure 3b). The color change in the HNB dye occurred more quickly than that of the AGE imaging, resulting in an improved detection efficiency. However, it is important to note that the change in the magnesium ion concentration indicates the presence of DNA amplification, but does not directly correlate with the amount of DNA amplified [26]. High concentrations of magnesium ions can significantly inhibit the amplification efficiency, while low concentrations can cause the amplification product to appear azure before the reaction, making it difficult to discern the color change and leading to false positives [27]. The detection of HNB dyes can be affected by the test equipment, which can reduce its accuracy. To reduce errors in such situations, RGB color analysis can be performed using Image J software (1.4.3.67). As depicted in Figure 3c, the negative control exhibits a similar proportion of the three colors. As the concentration of the bacterial fluid increases, the proportion of red gradually decreases, while the gap with the other two colors gradually widens. RGB color analysis allows us to differentiate between negative and positive results, thus helping to meet the qualitative requirements.

Figure 3b illustrates that the T line color decreases as the concentration of the bacterial solution decreases. Even at a concentration of 100 CFU mL^−1^, the detection line color was still different from that of the negative control. The light intensity of the T line on the ICTs was measured using ImageJ software. The detection limit of 5.7 × 10^0^ CFU mL^−1^ was significantly different from that of the negative control (NC), as shown by the light intensity data (Figure 3d). This suggests that the LAMP-ICTs may have a wider detection range compared to the colorimetric LAMP. The intensity of the T line was linearly related to the concentration of the corresponding bacterial solution in the range of 5.7 × 10^6^–5.7 × 10^0^ CFU mL^−1^, as shown in the calibration curve in Figure 3e. The fitting result was Y = 1044.3 lg(X) + 14925 (R^2^ = 0.9953), where Y represents the intensity of the T line and X represents the concentration of the bacterial solution. It should be noted that the semiquantitative analysis of the assay results is based on the light intensity of the T line in the ICT image of the subject. This study found that the analytical sensitivity achieved through the ICT assay was consistent with the results obtained from the AGE assay and the colorimetric LAMP analysis. The limit of detection was found to be 5.7 × 10^0^ CFU mL^−1^ (Figure 3a). The sensitivity was increased by 100-fold when compared to the real-time fluorescence PCR and PCR-LFD assays for the pure cultures [28,29]. Concurrently, the PCR cycle amplification process necessitated a considerable duration of approximately 1–2 h. In contrast, the LAMP amplification time in this study was only 40 min, which significantly reduced the detection time. Compared to other LAMP and LAMP-LFD assays, this assay has a sensitivity that is 10 times higher [23,30]. The study found that the sensitivity of the pure culture was equivalent to that of the CRISPR/Cas12a assay [31]. The data demonstrate that our established method is highly sensitive and has significant potential for the detection of *E. coli O157: H7* in food products.

### 3.8. Analysis Performance in Milk Samples

**Figure 4:**
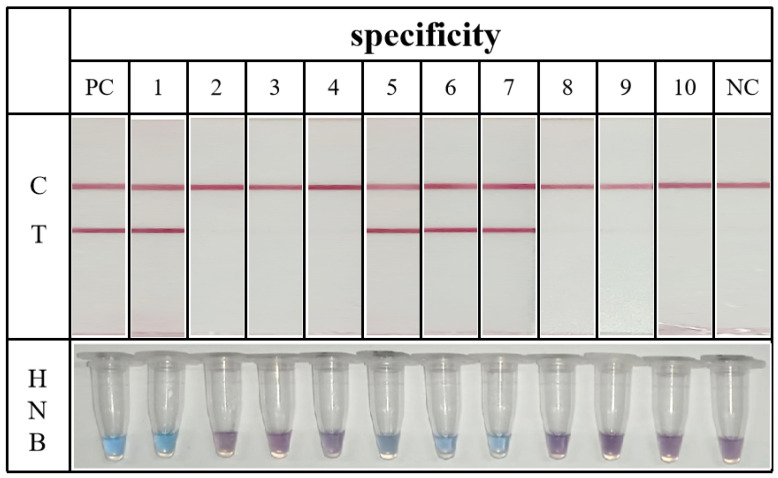
Specific detection in milk samples. The numbers 1–10 correspond to the addition of different strains of bacteria in Table 2, respectively.

The composition of food matrices is complex, particularly in animal-derived samples where the fat and protein components can significantly affect the test results. Different types of pathogenic bacteria can contaminate milk samples, leading to varying levels of contamination. To address this, we set up the contamination mode of different kinds of pathogenic bacteria. The specific experimental design is outlined in Table 2. The colorimetric LAMP method has a relatively short assay time. After the addition of the dye, the target strain assay tube turns blue or dark blue, while, in the case of the mixed contamination of other strains, the dye remains purple (Figure 4). The LAMP-ICT method can detect target strains in all cases of mixed contamination, regardless of the food matrix and stray bacteria. Additionally, false-positive results with the T line coloration do not occur when mixed with other pathogenic bacteria (Figure 4). The two established visualization methods are unaffected by stray bacteria and food matrices, demonstrating good specificity.

**Figure 5:**
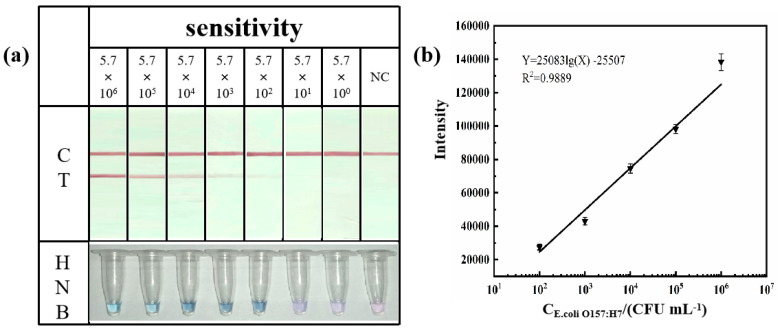
Sensitivity test in the sample (**a**) and standard curve of the LAMP-ICTs (**b**).

The limit of detection (LOD) for the colorimetric LAMP and LAMP-ICT methods in artificially inoculated milk samples was determined to be 5.7 × 10^2^ CFU mL^−1^ (Figure 5), which yielded the same results as the AGE sensitivity. The sensitivity experiments on the LAMP-ICT samples were analyzed, and the gray values of the T line were found to be linear within the detectable range (see Figure 5b). The fitted curve is Y = 25083 lg(X) − 25507 (R^2^ = 0.9889). The sensitivity of the food samples was lower than that of the bacterial fluids. It is possible that some proteins and lipids from the sample matrix remain in the supernatant after the high-temperature water bath, which can impact the specific binding of antigens and antibodies, ultimately affecting the sensitivity and stability of the immunoassay method. Table 3 provides comparisons with other reported detection methods for *E. coli O157: H7*. The table lists and compares the assay categories, detection times, and detection limits. The comparative results indicate that both visualization methods have short detection times, produce visible results, and possess good field detection capabilities. These characteristics make them suitable for the rapid field detection of *E. coli O157: H7* in remote pastures.

To assess the accuracy of the LAMP-ICTs in food samples, milk of different concentrations was purchased from local markets for the spiking recovery experiments (Table 4). The milk samples were fully packaged, sterilized, and tested prior to the sale, ensuring that the milk samples spiked with zero were free of bacteria. The recovery values for the *E. coli O157: H7* ranged from 91.1% to 103.5%, with relative standard deviations (RSDs) between 5.7 and 8.38%, all of which were less than 10%. These results demonstrate the accuracy of the designed strategy and its potential for preliminary use in detecting *E. coli O157: H7* in milk.

## 4. Conclusions

This study presents the development of two visual LAMP methods, namely, the colorimetric LAMP and LAMP-ICTs, for the rapid detection of *E. coli O157: H7*. The primers used in the study were newly designed. The reaction speed and sensitivity of detecting *E. coli O157: H7* were improved by adding a set of loop primers (LBs) to the designed primers. These analytical methods were able to distinguish *E. coli O157: H7* from other common pathogenic species, and its analytical sensitivity was consistent with the results of the AGE, with a detection limit of 5.7 × 10^0^ CFU mL^−1^. Both methods are expected to be used in real food samples, with a sensitivity of 5.7 × 10^2^ CFU mL^−1^ in milk samples. Additionally, the recoveries of the LAMP-ICTs in the milk ranged from 91% to 103%, with RSDs of less than 10%.

The colorimetric LAMP does not require waiting, and the results can be observed directly after the water bath. However, the color change exhibited instability during the microdetection, making it difficult to distinguish between non-specific amplification and true-positive signals. Compared to the colorimetric LAMP method, the LAMP-ICT method enables quantitative detection with a high specificity and stability. It is also more accurate, without any experimental results that may cause confusion for testers. By changing the functional primer set, both methods can also detect other bacteria. This will benefit public health by enabling the rapid diagnosis of pathogenic bacteria and reducing foodborne illness outbreaks.

## Figures and Tables

**Table 1:** List of primers used in this study.

Gene	Primer Name	Sequence (5′-3′)		5′-Labeled	Reference
*rfbE*	E1	F3	CCACAAGGAAAGTAAAGATGTT		[23]
		B3	CCAACCAAGATCCTCAGC		
		FIP	CAAGGTGATTCCTTAATTCCTCTCTACACTTATTGGATGGTCTCA	digoxin	
		BIP	AACTCATCGAAACAAGGCCAGGTGCTTTTGATATTTTTCCGAGTA	biotin	
	E2	F3	TGGAATGGTTGTCACGAA		This study
		B3	GCGATTTCACGTTTTCGT		
		FIP	TTGCCTATGTACAGCTAATCCTTGTTTTTGACAAAACACTTTATGACCG	digoxin	
		BIP	ATTGGCATGACGTTATAGGCTACTTTTGCTTGTTCTAACTGGGCTAA	biotin	
*stx2*	21	F3	TCGGTGTCTGTTATTAACCA		[24]
		B3	TGGAAACCGTTGTCACA		
		FIP	AGACGAAGATGGTCAAAACGCGCAGTTATTTTGCTGTGGA	digoxin	
		BIP	CCGGGTTCGTTAATACGGCACGGGCACTGATATATGTGT	biotin	
	22	F3	CTGCTGTGACAGTGACAA		This study
		B3	ACAACGGTTTCCATGACAA		
		FIP	TCATCATATCTGGCGTTAATGGAGTTTTCTGCTCTGGATGCATCTC	digoxin	
		BIP	AACCAGTGAGTGACGACTGATTTTCGGACAGCAGTTATACCA	biotin	
		LB	TTCCGGAACGTTCCAGCG		

**Table 2:** Experimental design for the sample specificity.

	*Salmonella*	*E. coli O157: H7*	*Listeria monocytosis*	*Staphylococcus aureus*	*Campylobacter jejuni*
1	+	+	−	−	−
2	+	−	+	−	−
3	+	−	−	+	−
4	+	−	−	−	+
5	−	+	+	−	−
6	−	+	−	+	−
7	−	+	−	−	+
8	−	−	+	+	−
9	−	−	+	−	+
10	−	−	−	+	+

+: addition of this bacteria; −: non-addition of this bacteria.

**Table 3:** Comparison with other *E. coli O157:H7* detection methods.

Technique	Sample Type	Time	Limit of Detection	Reference
Fluorescence ELISA	Milk	~5 h	3.75 × 10^3^ CFU mL^−1^	[13]
Colorimetric aptasensor	Milk	250 min	2.3 × 10^3^ CFU mL^−1^	[32]
PCR+LFS	Carrot	60 min	10^2^ CFU g^−1^	[33]
qPCR	Ground beef	~4 h	10^1^ CFU mL^−1^	[34]
Photoelectrochemical biosensor	Milk, beef, fish	~40 min	5 CFU mL^−1^	[35]
Smartphone-based LAMP	Milk	4 h	10^1^ CFU mL^−1^	[36]
LAMP+DMF	Spiked milk	50 min	10^3^ CFU mL^−1^	[37]
LAMP-LFD	Lettuce	~2 h	10^1^ CFU mL^−1^	[23]
Colorimetric LAMP	Milk	40 min	5.7 × 10^2^ CFU mL^−1^	This study
LAMP-ICTs	Milk	50 min	5.7 × 10^2^ CFU mL^−1^	This study

**Table 4:** LAMP-ICT test for *E. coli O157: H7* spike recovery experiments in milk samples.

Group	Before (CFU mL^−1^)	Add (CFU mL^−1^)	After (CFU mL^−1^)	Recovery ^a^ (%)	RSD ^a^ (%)
1	0	0	ND	-	-
2	0	5.7 × 10^2^	519 ± 36	91.1	6.94
3	0	5.7 × 10^3^	5670 ± 475	99.5	8.38
4	0	5.7 × 10^4^	58,979 ± 3359	103.5	5.70

^a^ n = 3; ND: no detection signal.

## Data Availability

The original contributions presented in the study are included in the article/Supplementary Materials, further inquiries can be directed to the corresponding authors.

## Supplementary Materials

The following supporting information can be downloaded at: https://www.mdpi.com/article/10.3390/foods13132143/s1, Figure S1: Primer screening results; Figure S2: LAMP system optimization results; Figure S3: Feasibility detection; Figure S4: Optimization of HNB dye concentration; Figure S5: Assay results for 10% NaCl; Figure S6: Optimization of ICT conditions; Figure S7: Specific detection in the bacterial solution.
