# Supplementary material for: Visual Loop-Mediated Isothermal Amplification (LAMP) Assay for Rapid On-Site Detection of Escherichia coli O157: H7 in Milk Products
Source: Foods. 2024 Jul 5;13(13):2143. doi: 10.3390/foods13132143 (PMC11241362; doi:10.3390/foods13132143)
Supplement: Supplementary file 1 [file foods-13-02143-s001.zip › foods-3031562-supplementary.pdf]

Supplementary Material

Visual loop-mediated isothermal amplification (LAMP) assay for rapid on-site detection of *Escherichia coli O157: H7* in milk products

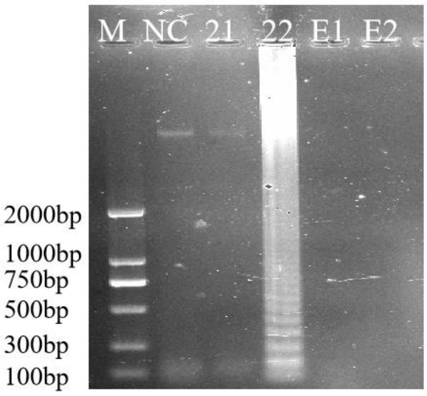

Figure S1. Primer screening results.

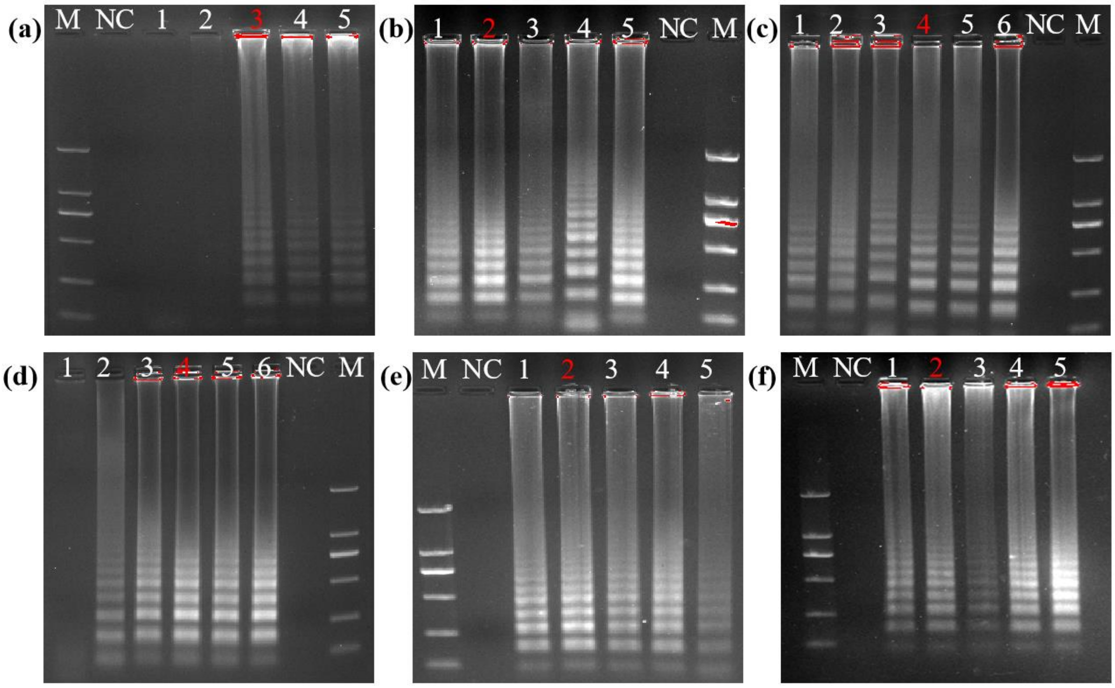

Figure S2. LAMP system optimization results. (a) Optimization of magnesium ion concentration. 1~5 were magnesium ion concentration of 2 μM, 4 μM, 6 μM, 8 μM, 10 μM, respectively. (b) Optimization of dNTPs concentration. 1~5 were dNTPs concentration of 0.8 μM, 1.2 μM, 1.6 μM, 2.0 μM, 2.4 μM, respectively. (c) Optimization of the ratio of the internal and external primer. 1 ~ 6 The ratios of the internal and external primer were 1:1, 2:1, 3:1, 4:1, 6:1, 8:1 (the external primer concentration was 0.2 μM.), respectively. (d) Optimization of Bst-DNA polymerase enzyme concentration. 1~6 concentration was 0.08 U/μL, 0.16 U/μL, 0.24 U/μL, 0.32 U/μL, 0.40 U/μL, 0.48 U/μL. (e) Optimization of reaction temperature. 1~5 Reaction temperatures were 61°C, 63°C, 65°C, 67°C, 69°C, respectively. (f) Reaction time optimization.1~5 reaction times were 35min, 40min, 45min, 50min, 55min, respectively.

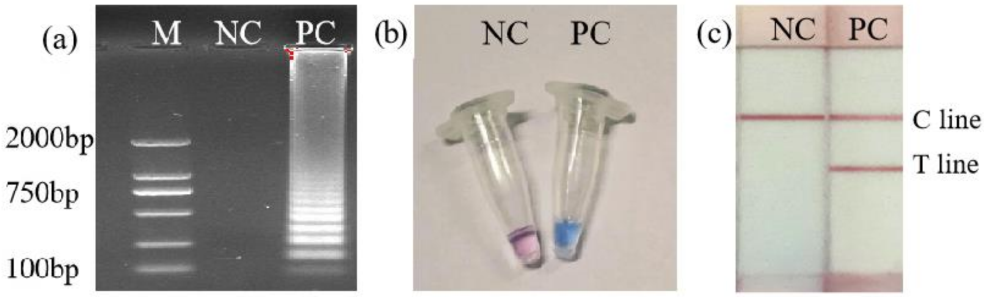

Figure S3. Feasibility detection.

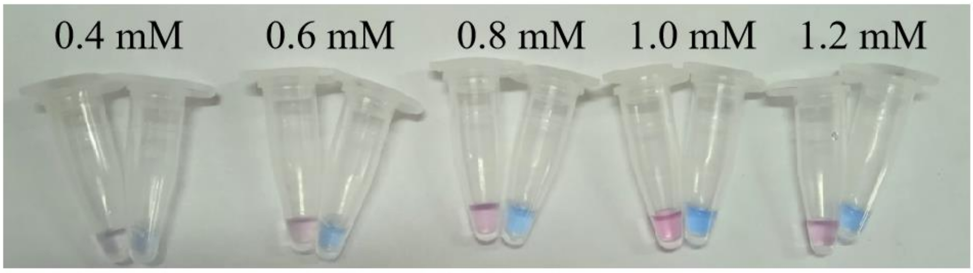

Figure S4. Optimization of HNB dye concentration.

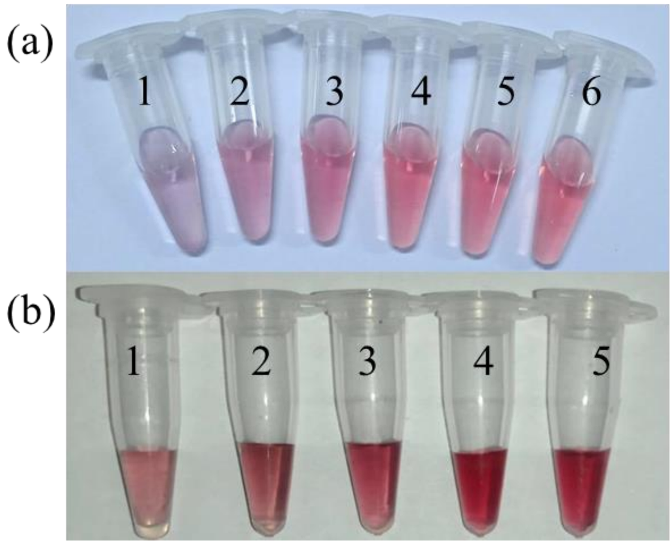

Figure S5. Assay results for 10% NaCl. (a) Optimization of the addition amount of 0.1 M K_2CO_3: 1 ~ 6 for 0, 2, 4, 6, 8, 10 μL. (b) Optimization of the addition amount of digoxin antibody: 1 ~ 5 for 2.5, 3.5, 4.5, 5.5, 6.5 μg.

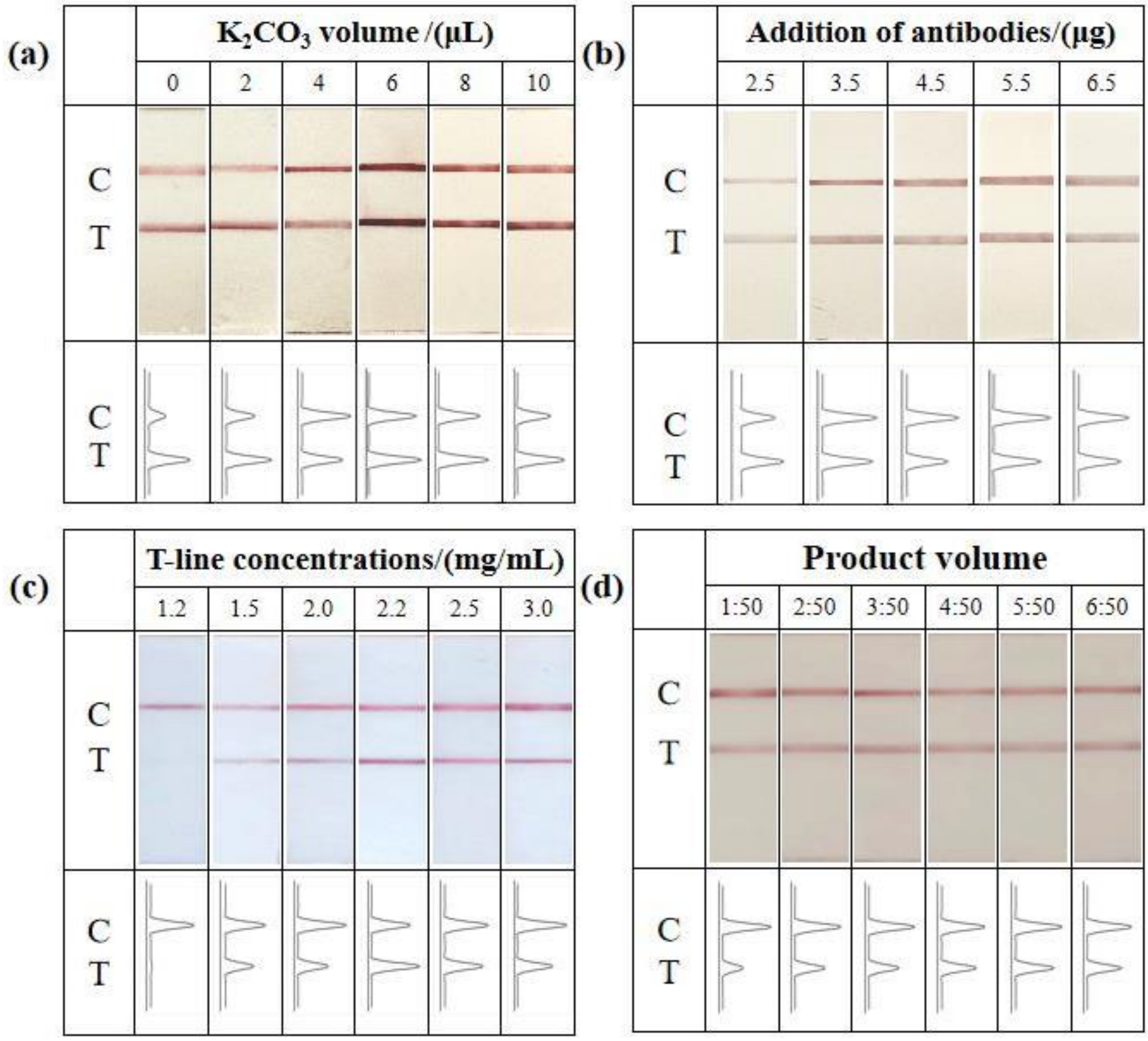

Figure S6. Optimization of ICTs conditions. (a) Optimization results of 0.1 mol L^{-1} K_2CO_3 addition volume. (b) Results of antibody addition volume optimization in gold-labeled probes. (c) Optimization results for the concentration of the T line inclusion principle. (d) Optimization results for different product spiking volumes. From left to right, LAMP product : 0.01M PBS buffer = 1:50, 2:50, 3:50, 4:50, 5:50, 6:50, 7:50.

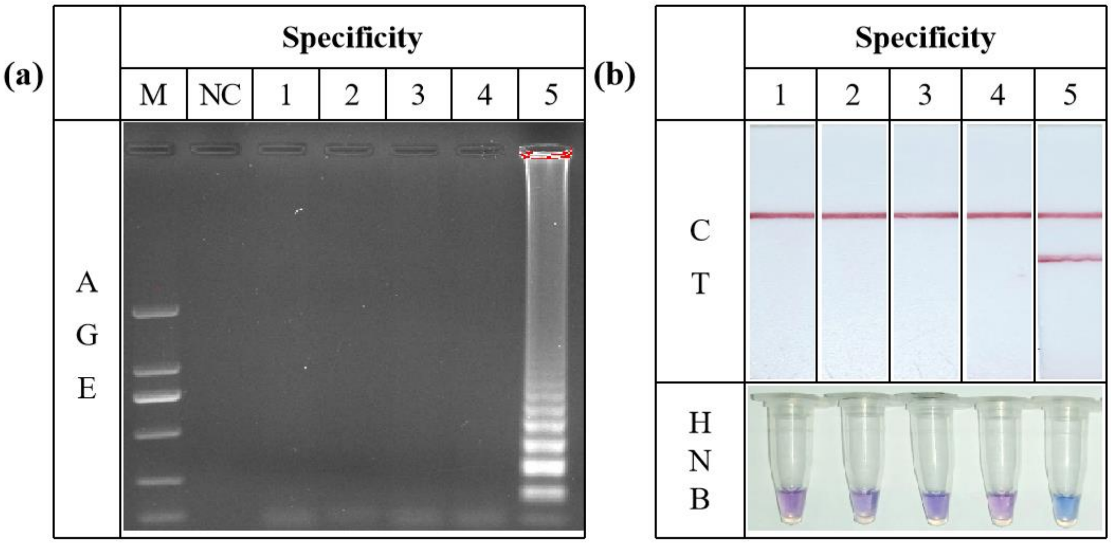

Figure S7. Specific detection in the bacterial solution. (a) AGE specificity assay. (b) LAMP-LFD specific assay and HNB specificity assay. M: DNA Marker2000, NC: Negative control, 1-5 for *Salmonella Typhimurium spp.* (ATCC14028), *Staphylococcus aureus* (ATCC25923), *Listeria monocytgenes* (ATCC19115), *Campylobacter jejuni* (BW180151), *E. coli O157:H7* (CICC21530).
